# Predictive learning: its key role in early cognitive development

**DOI:** 10.1098/rstb.2018.0030

**Published:** 2019-03-11

**Authors:** Yukie Nagai

**Affiliations:** National Institute of Information and Communications Technology, Suita, Osaka 565-0871, Japan

**Keywords:** predictive learning, predictive coding, developmental robotics, social cognitive development, autism spectrum condition

## Abstract

What is a fundamental ability for cognitive development? Although many researchers have been addressing this question, no shared understanding has been acquired yet. We propose that predictive learning of sensorimotor signals plays a key role in early cognitive development. The human brain is known to represent sensorimotor signals in a predictive manner, i.e. it attempts to minimize prediction error between incoming sensory signals and top–down prediction. We extend this view and suggest that two mechanisms for minimizing prediction error lead to the development of cognitive abilities during early infancy. The first mechanism is to update an immature predictor. The predictor must be trained through sensorimotor experiences because it does not inherently have prediction ability. The second mechanism is to execute an action anticipated by the predictor. Interacting with other individuals often increases prediction error, which can be minimized by executing one's own action corresponding to others’ action. Our experiments using robotic systems replicated developmental dynamics observed in infants. The capabilities of self–other cognition and goal-directed action were acquired based on the first mechanism, whereas imitation and prosocial behaviours emerged based on the second mechanism. Our theory further provides a potential mechanism for autism spectrum condition. Atypical tolerance for prediction error is hypothesized to be a cause of perceptual and social difficulties.

This article is part of the theme issue ‘From social brains to social robots: applying neurocognitive insights to human–robot interaction’.

## 1. Introduction

Human infants acquire various types of cognitive abilities after birth. Although neonates do not seem to inherently know how to control their body or how to interact with an environment, their actions become more accurate and purposeful with increased experience. The ability to communicate with other individuals also develops through primary social interactions. Young infants, who may not know with whom to interact (e.g. social versus non-social agents) or how to interact with them, learn to be engaged in social relationships with the help of their carers. A big mystery here is the nature of the fundamental ability that leads to cognitive development. Despite a number of findings from behavioural and neuroscience studies, the mechanisms underlying cognitive development have not yet been completely uncovered. One well-known developmental theory is the dynamical systems approach [1,2], which suggests that motor and cognitive development appears as a dynamical change within a complex system. For example, new behaviours are thought to emerge as a result of many decentralized and local interactions between infants and their environment. This theory provides a feasible explanation; however, it focuses on the phenomenal aspect rather than the underlying neural mechanism. It is therefore hard for computational researchers to break the theory down into mathematical architectures. An open challenge is to devise a computational unified theory that accounts for the underlying mechanisms of development.

Robotics researchers have been addressing the above issue by synthesizing infant-like development in robots [3–5]. Cognitive abilities that have been successfully reproduced in robots include self–other cognition [6–8], imitation [9,10], joint attention [11–14], intrinsic motivation [15,16] and so on. Their experiments have empirically supported conceptual theories proposed by psychologists and, moreover, revealed new perspectives to cognitive development. For example, sensorimotor contingency, which has been viewed in psychology as a core mechanism for development [17,18], enabled robots to acquire even social behaviours based on non-social sensorimotor experiences (e.g. [11,14,19]). However, these studies target only specific cognitive functions such as joint attention. The extent to which these architectures can account for cognitive development has not been determined yet.

This paper aims to propose a computational theory that explains and replicates the diversity and continuity in cognitive development. Inspired by recent evidence from neuroscience studies, we suggest that predictive learning of sensorimotor signals plays a key role in early cognitive development. It is known that the human brain has an internal model of the world [20]. Humans are able to simulate the dynamics of an environment and to purposefully control their body by employing the internal model. Researchers in cognitive neuroscience have advocated that predictive coding accounts for the internal model [21–23]. They suggest that the brain attempts to match incoming sensory signals with top–down prediction by minimizing prediction error. Computational models based on predictive coding have also been proposed. Rao & Ballard [24] showed that top–down prediction in a visual cortex model led to the development of simple-cell-like receptive fields. Tani and colleagues [25,26] demonstrated action learning in robots. Their robots, which were equipped with a recurrent neural network, learned to minimize prediction error and thereby acquired the ability to manipulate objects. Their close analysis even revealed that motor behaviours were developmentally structured in the neural network, the process of which was analogous to infant development [27,28]. Following these successful studies, this paper presents a unified computational account for cognitive development. We suggest that two mechanisms for minimizing prediction error lead to the development of cognitive behaviours: updating an immature predictor to refine own sensorimotor abilities and executing an action estimated by the predictor in response to others’ action. Our experiments using robotic systems demonstrate how cognitive abilities such as self–other cognition, goal-directed action and helping behaviour develop based on the above mechanisms.

The remainder of this paper is organized as follows: §2 first presents a basic architecture of predictive learning. A traditional theory of predictive coding is extended to more throughly explain cognitive development. Then, our theory for cognitive development is proposed, where the advantages of predictive learning over contingency learning are explained. Section 3 provides case studies of robot experiments. Our computational models reproduce infant-like developmental dynamics in robots. Section 4 discusses further potentials of our theory. We suggest that developmental disorders such as autism spectrum condition (hereafter ASC) can be explained by atypicality in predictive learning. Finally, profound discussion and conclusion are given in §§5 and 6, respectively.

## 2. Predictive learning theory accounting for early cognitive development

### (a) Basic architecture of predictive learning

**Figure 1.**
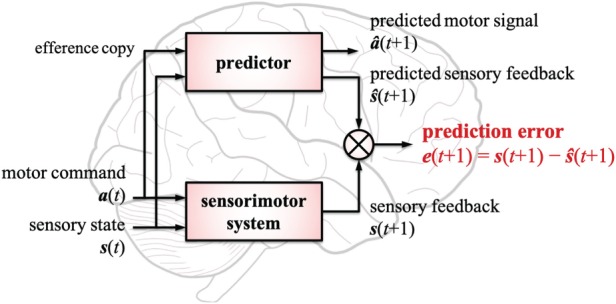
Basic architecture for sensorimotor predictive learning that consists of two modules: a sensorimotor system (the lower box) and a predictor (the upper box). The predictor works as an internal model of the sensorimotor system; it learns to estimate sensorimotor signals, $\hat{s}(t+1)$ and $\hat{a}(t+1)$, at time *t* + 1, while the sensorimotor system actually executes an action a(t) under a sensory state s(t) at *t* and consequently receives a sensory feedback s(t+1) from the environment. Predictive learning aims to minimize prediction error e(t+1), which is calculated as the difference between s(t+1) and $\hat{s}(t+1)$. (Online version in colour.)

Figure 1 illustrates a basic architecture of predictive learning that is modified from [20,29]. The architecture consists of two modules: a sensorimotor system (the lower box), which interacts with the environment, and a predictor (the upper box), which simulates the sensorimotor system in the brain.

The first module is the sensorimotor system, which corresponds to the body. It has the roles of executing an action $a(t)={\left(a_{1}(t),a_{2}(t),\ldots ,a_{N_{a}}(t)\right)}^{T}$ under a certain sensory state $s(t)={\left(s_{1}(t),s_{2}(t),\ldots ,s_{N_{s}}(t)\right)}^{T}$ at time *t* and of consequently perceiving a sensory feedback s(t+1)= ${\left(s_{1}(t+1),s_{2}(t+1),\ldots ,s_{N_{s}}(t+1)\right)}^{T}$ at *t* + 1 from the environment. *N*_*a*_ and *N*_*s*_ indicate the number of action modalities (e.g. gaze, speech and hand movement) and of sensory modalities (e.g. visual, audio, tactile and proprioceptive senses) that the body has, respectively. For example, if an infant extends his arm *a*_*i*_(*t*) (*i* = arm movement) towards an object *s*_*j*_(*t*) (*j* = visual sense), the sensorimotor system receives a tactile signal *s*_*k*_(*t* + 1) (*k* = tactile sense) as well as a visual feedback *s*_*j*_(*t* + 1) when he reaches the object. How to segment sensory and action modalities and how to discretize time depend on target scenarios.

The second module is the predictor, which represents the internal model of the sensorimotor system. The predictor receives s(t) and the efference copy of a(t) in order to estimate both a sensory state $\hat{s}(t+1)$ and a motor signal $\hat{a}(t+1)$ at *t* + 1, while the sensorimotor system affects the environment. The aim of the predictor is to accurately simulate the sensorimotor system by learning to minimize prediction error e(t+1), which is calculated as the difference between the actual sensory feedback s(t+1) and the predicted one $\hat{s}(t+1)$:

2.1 $$e(t+1)=s(t+1)-\hat{s}(t+1).$$

Note that the predictor can have a hierarchical structure to calculate and minimize many different levels of prediction error, although figure 1 illustrates the predictor as a box for the sake of simplicity. The prediction error usually appears to have a larger value in the early stage of development and is gradually minimized through sensorimotor learning. We suggest that this learning process and the process of executing the predicted action to minimize prediction error produce various cognitive behaviours ranging from non-social to social ones.

### (b) Cognitive development based on sensorimotor predictive learning

**Figure 2.**
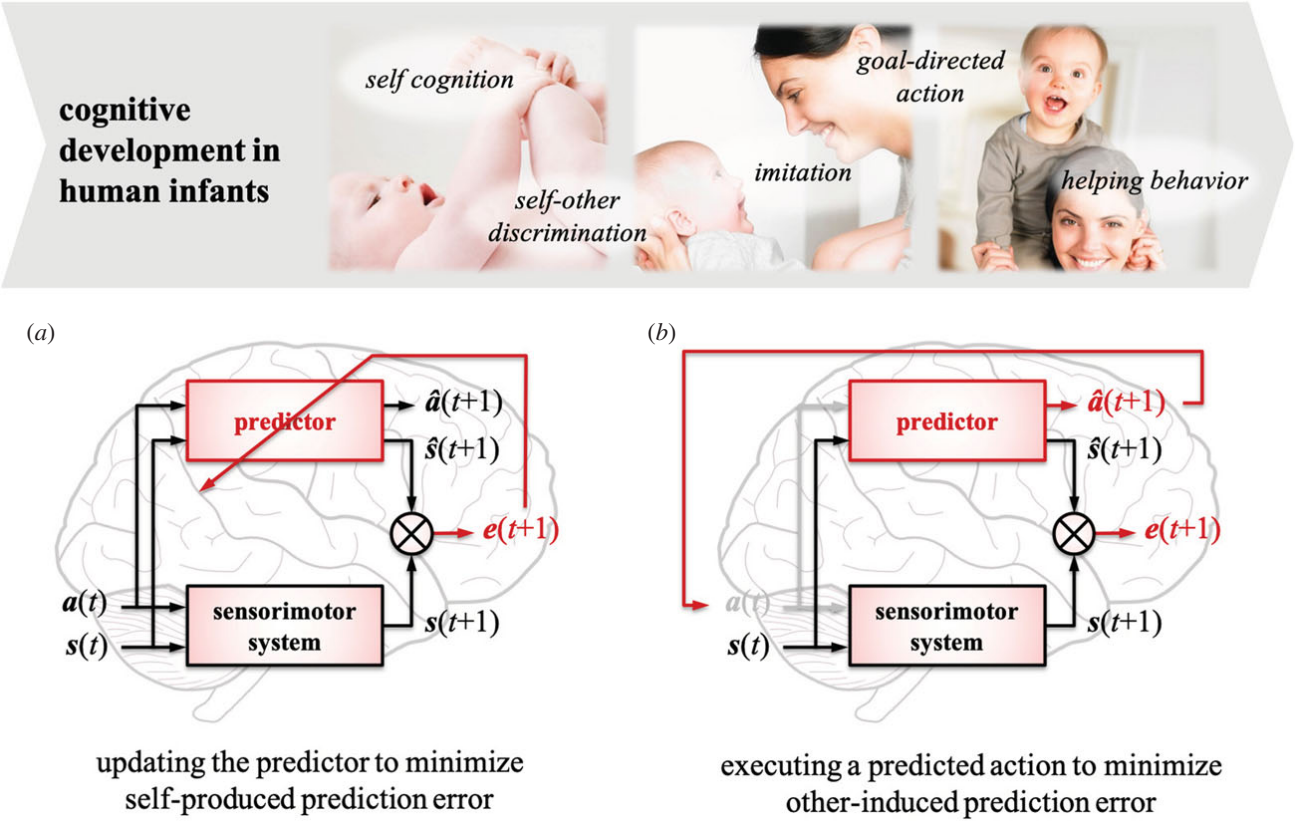
Examples of cognitive abilities infants acquire in the first few years of life (top) and two mechanisms for predictive learning as underlying mechanisms for development (bottom). The first mechanism ((*a*) at the bottom left) is to update the predictor through the minimization of prediction error e(t+1), where an increase in e(t+1) is mainly caused by the immaturity of the predictor. This mechanism enables infants to acquire the abilities of self-cognition, self–other discrimination, goal-directed actions etc. The second mechanism ((*b*) at the bottom right) is the execution of a predicted motor command $\hat{a}(t+1)$ to minimize e(t+1). In this case, an increase in e(t+1) is mainly caused by a lower predictability of others’ actions. Minimization of the error thus leads to emergence of social behaviours such as imitation and helping actions. (Online version in colour.)

Figure 2 presents our theory of cognitive development: the top shows examples of cognitive behaviours to appear during early infancy, and the bottom illustrates two architectures for minimizing prediction error. Target behaviours include self–other cognition, goal-directed action, helping behaviour and so on, which are assumed to be acquired through sensorimotor learning. Higher cognitive abilities such as language use and decision-making are not yet incorporated in our current theory, which is discussed in more detail in §5. Of importance here is that despite different abilities appearing in infancy, they share a common mechanism based on predictive learning. The first mechanism shown in figure 2*a* is to update the predictor by minimizing self-produced prediction error, and the second mechanism shown in figure 2*b* is to execute a predicted action to minimize other-induced prediction error. Note that the two mechanisms are interlinked during development, although they are separately illustrated.

#### (i) Updating the predictor to minimize self-produced prediction error

The first mechanism of predictive learning aims to update the predictor through sensorimotor experiences (figure 2*a*). Human infants are born with an immature predictor that produces a higher prediction error e(t+1) for all events. Although they show reflexes and body babbling as inherent abilities, a primary predictor cannot yet accurately anticipate the sensory outcomes. For example, infants at this stage would not even know how sensory signals represent their body (i.e. body image and body scheme [30,31]) or how the body is segmented from the environment. Our theory suggests that updating the predictor through the minimization of e(t+1) enables infants to first recognize their body as sensory signals producing no or very small prediction error. Neonates, who are not supposed to have the ability to recognize their body yet, often gaze at their hands and feet, and even put them into their mouth, which provides multimodal perceptions of their body. These experiences allow them to refine the predictor by associating the executed action a(t) with the resultant state change, i.e. the change from s(t) to s(t+1). Because an infant's own body usually reacts to his/her own action in a highly contingent manner, its prediction error e(t+1) is expected to approach zero over learning. Therefore, the ability to recognize one's own body is thought to develop through the refinement of the predictor.

The above process also leads to the development of self–other discrimination [32–34]. Other individuals are recognized as weakly predictable entities—but not unpredictable owing to social relationship—whereas infants’ own bodies are perceived as highly predictable sensory signals after learning. Of interest here is that others’ behaviours are slightly beyond prediction but not too far from prediction in social contexts. Because they share the same environment with infants, their internal models are expected to be similar to those of infants, which produces moderate prediction error. We suggest that moderate prediction error continuously motivates infants to communicate with and learn about others to further minimize the error. Non-social objects, in contrast, exhibit different dynamics. Their predictability changes more drastically between predictable and unpredictable. Toys, for example, become highly predictable while being manipulated, whereas they are less predictable otherwise, or *vice versa* depending on their property. Neuroscience studies support this implication; the brains of monkeys appear to represent a tool in manipulation as a part of their body [35,36]. It is presumed that no or a little prediction error generated by a manipulated tool allowed monkeys to extend their body representation. We suggest that infants come to be able to discriminate the self, other individuals (i.e. social entities) and objects (i.e. non-social entities) based on their predictabilities.

Other cognitive abilities that develop through the process of updating the predictor include goal-directed actions [37–39]. Reaching for an object, manipulating it, and so on are considered as goal-directed. Piaget [40] proposed six stages of sensorimotor development, in which the first three stages illustrate how infants acquire goal-directed actions. The first stage consists of simple reflexes. Infants from birth to one month are endowed only with limited capabilities such as reflexes (e.g. sucking and grasping). Their predictor cannot yet simulate the sensorimotor system, and thus infants of this age do not show any goal-directed actions. Reflexes, however, allow infants to accumulate sensorimotor experiences to refine the predictor. Their behaviour becomes accurate and intentional during the second and third stages of development, which correspond to 1–4 and 4–8 months old, respectively. These stages are named primary and secondary circular reactions according to Piaget [40], in which infants start reproducing actions that happened by accident but brought interesting outcomes. Infants at these stages seem to actively learn the predictor such that it properly simulates interesting events caused by their actions. As a consequence of this process, the predictor becomes able to intentionally produce actions induced by the current sensory state, which is observed as goal-directed actions.

#### (ii) Executing an action to minimize other-related prediction error

The second mechanism of predictive learning is to execute a motor signal $\hat{a}(t+1)$ estimated by the predictor (figure 2*b*). The predictor is employed not only to simulate one’s own action but also to predict actions performed by other individuals. In this case, the prediction error e(t+1) appears as a higher value even after learning, because behaviours of other individuals are less predictable as explained before. We suggest that primary forms of social behaviours emerge as a result of an execution of $\hat{a}(t+1)$ to minimize e(t+1). When infants observe other individuals performing an action, the action and its outcome are perceived as s(t) and s(t+1), respectively. In parallel with this, the predictor estimates a sensory state $\hat{s}(t+1)$ and a corresponding motor signal $\hat{a}(t+1)$ based on s(t). Note that no action is executed by infants at *t* (see a(t) with diminished colour in figure 2*b*). An important assumption here is the correspondence between the self and others (i.e. the mirror neuron system) represented in the predictor. As the predictor is trained through the development of self–other cognition, it cannot differentiate the self from others in the early stage of development. The assimilated representation of the self and others, however, enables infants to recognize the observed actions performed by others as if these were their own actions. Executing the predicted action $\hat{a}(t+1)$ is thus expected to minimize e(t+1).

We suggest that primary forms of social behaviours such as imitation and helping actions emerge based on the above mechanism. An increase in e(t+1) triggers execution of $\hat{a}(t+1)$ to minimize e(t+1). Imitative behaviours are generated as soon as infants detect an increase in e(t+1). Helping actions are also produced when infants detect a larger increase in e(t+1). If other individuals fail in achieving a goal, e(t+1) starts increasing because of the discrepancy between the predicted goal state $\hat{s}(t+1)$ and the actual state s(t+1). Therefore, infants try to minimize e(t+1) by executing their own action $\hat{a}(t+1)$, which results in the predicted goal state $\hat{s}(t+1)$. The infants’ behaviour then looks as if they help others achieve the goal although the infants do not have such an intention. Our theory indicates that infants’ social behaviour originates from the minimization of prediction error (i.e. non-social motivation) rather than from social motivation.

### (c) Predictive learning versus contingency learning

**Figure 3.**
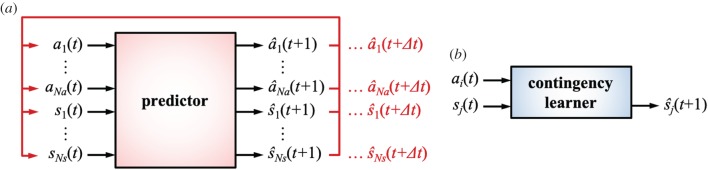
Predictive learning versus contingency learning. (*a*) Predictive learning with multistep and multimodal prediction. (*b*) Contingency learning with single-step and single-modal prediction. (Online version in colour.)

We emphasize advantages of predictive learning over contingency learning. Contingency learning has been suggested to play an important role in infant development [17–19], and computational models inspired by this view have been proposed to enable robots to develop like infants (e.g. [11,14]). We, however, suggest that predictive learning, which extends the concept of contingency learning, provides a more general and powerful architecture for cognitive development. Their differences are illustrated in figure 3, where two advantageous characteristics of predictive learning are highlighted: *multistep* prediction and *multimodal* prediction.

#### (i) Multistep prediction

The first advantage of predictive learning is multistep prediction. Whereas contingency learning usually targets single-step prediction (i.e. *t* → *t* + 1), predictive learning is able to estimate multistep signals (i.e. *t* → *t* + 1 → ··· → *t* + Δ*t*) by concatenating single-step prediction. Figure 3*a* depicts a predictor, where the predicted signals are recurrently fed back to the predictor and finally the signals at *t* + Δ*t* are obtained as output. A key mechanism that achieves this recurrent prediction is the estimation of motor signals. As the predictor learns to anticipate both the sensory $\hat{s}(t+1)$ and motor signals $\hat{a}(t+1)$, they can be used as the imaginary input for the next time step *t* + 1. The output signals $\hat{s}(t+2)$ and $\hat{a}(t+2)$ are then used for further prediction, which results in multistep prediction. By contrast, a contingency learner shown in figure 3*b* learns to estimate only ${\hat{s}}_{\;j}(t+1)$ but not ${\hat{a}}_{i}(t+1)$. Sensorimotor contingency was originally defined as lawful regularities between an executed action *a*_*i*_(*t*) and a sensory change from *s*_*j*_(*t*) to ${\hat{s}}_{\;j}(t+1)$ [41–43]. Although it is possible to extend contingency learning to deal with multistep prediction (e.g. [43,44]), predictive learning provides a consistent explanation from non-social to social sensorimotor development, as described in figure 2.

Developmental studies supporting the importance of multistep prediction have been reported. For example, older infants exhibit the ability to predict the goal of others’ action quicker than younger infants [45,46]. Our computational study demonstrated that this development can be achieved by extension of prediction length, which is triggered by improvement in learning accuracy [47,48]. In the early stage of development, our system made fewer steps of prediction because of their lower prediction accuracy. Its resultant behaviours were thus similar to those observed in younger infants. The system then gradually increased the concatenation of prediction as learning progressed, which produced behavioural development as observed in infants. Limited capability of ASC to interpret others’ intention [49–51] might also be attributed to their limited capacity for prediction. A detailed computational account thereof is given in §4.

#### (ii) Multimodal prediction

The second advantage of predictive learning is multimodal prediction. As shown in figure 3*b*, a contingency learner normally deals with a single-modality signal according to the original definition [41–43]. A relationship between the *i*-th motor signal and the *j*-th sensory signal is represented in a contingency learner although it can also be extended to deal with multimodal signals. A predictor, by contrast, can learn to estimate multimodal signals as depicted in figure 3*a*, where (1, …, *N*_*a*_) and (1, …, *N*_*s*_) denote action and sensory modalities, respectively. All the signals are integrated in the predictor so as to mutually affect their prediction. That is, a signal ${\hat{s}}_{\;j}(t+1)$ is estimated based not only on *s*_*j*_(*t*) from the same modality but also on *s*_*k*_(*t*) (*k* ≠ *j*) from different modalities. This mutual prediction occurs between motor and sensory prediction as well as within each prediction.

The ability of multimodal prediction is also observed in infants, which is often termed multimodal integration. For example, infants can associate speech with the mouth movement of a person producing the speech (i.e. audio and visual integration) [52] and visually recognize an object explored by their mouth (i.e. visual, tactile and proprioceptive integration) [53]. The McGurk effect, which is a well-known phenomenon demonstrating the interaction between hearing a voice and seeing a person speaking, is also observed in older infants [54]. This evidence supports the advantage of multimodal predictive learning.

## 3. Case studies of computational modelling for robot cognitive development

This section presents case studies of computational modelling to support our theory. We previously demonstrated that robots equipped with predictive learning could acquire the capabilities of self–other cognition [55,56], goal-directed actions [57], helping behaviours [58] and so on. The reasons for choosing these cognitive functions include the high level of interest that they have generated in developmental psychology, much behavioural evidence to compare with computational models, and their developmental diversity ranging from non-social to social cognition. It is also important to note that the development of these functions can be achieved by a common mechanism of predictive learning, although they seem to be less interconnected at behavioural levels. The details of these studies are described in papers [55–58], and only the key ideas and key findings of these studies are presented here to support out theory. Refer to the relevant papers for the details.

### (a) Development of self–other cognition

The ability to recognize the self and others develops through the update of an immature predictor, which corresponds to the first mechanism of predictive learning (figure 2*a*). One’s own actions are highly predictable, whereas others’ actions are less predictable. Hence, the predictor comes to be able to differentiate the sensory signals related to own actions and those related to others based on their prediction error. An important implication here is that a function such as the mirror neuron system emerges as a by-product of the above development. The predictor is expected to acquire the ability to predict not only one’s own sensorimotor sequences but also motor signals corresponding to observed actions generated by others.

**Figure 4.**
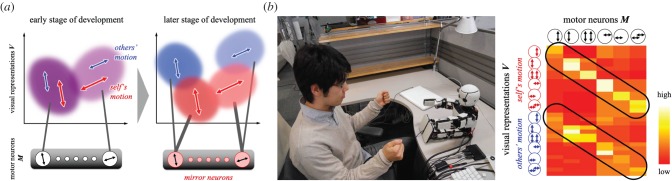
Development of self–other cognition based on predictive learning (adapted from [55,56]). (*a*) A computational model for the development of self–other cognition. Visual representations ***V*** are associated with motor neurons ***M***, while the acuity of visual perception improves over development. This development allows a robot to learn the equivalence between its own motions and others’ motions through sensorimotor predictive learning. (*b*) An experimental setting (left) and a result of sensorimotor learning (right). The robot acquired stronger associations between motor neurons and visual representations corresponding to both its own and others’ motions. These associations serve the function of mirror neuron systems. (Online version in colour.)

Figure 4*a* depicts our computational model proposed in [55,56]. A robot learned a predictor by associating motor neurons M (bottom) with visual representations V (top) through interactions with a carer (see the image in figure 4*b* (left) for the experimental setting). The model employed associative learning in order to minimize prediction error. We suggest that associative learning be regarded as a type of predictive learning because both architectures acquire regularities in sensorimotor coordination. In the early stage of development, the robot had lower perceptual acuity similar to infants. Hand movements produced by the robot and by the carer could not be differentiated in the visual space despite their inherent differences with respect to time (i.e. temporal delay) and space (i.e. position in the robot’s camera image). As seen in figure 4*a* (left), visual clusters containing both the robot’s motion and the corresponding carer’s motion were associated with the relevant motor neurons because differences between them were not detectable. In the later stage of development, the robot improved in visual acuity and therefore differentiated visual clusters into two: one for the robot and the other for the carer. As seen in figure 4*a* (right), differentiated clusters came to be associated with the same motor neuron because they were originally contained in the same cluster. This developmental change from self–other assimilation to self–other discrimination enabled the robot to acquire a function such as that of the mirror neuron system. The experimental result shown in figure 4*b* (right) supports this. The association between M (the columns) and V (the rows) exhibited higher values along the two diagonal axes, which represented the same correspondence between the self and other as that in the mirror neuron system. Note that this representation was acquired only when the robot had perceptual development. Perceptual immaturity in the early stage of development enabled the robot to detect the self–other correspondence even if the carer did not always imitate the robot.

Our result sheds light on debates on the origin of the mirror neuron system. Meltzoff and Moore [59,60], on the one hand, proposed a hypothesis they termed active intermodal mapping. They suggest that infants are endowed with supramodal representation in their brain, where the equivalence between the self and others perceived in different modalities is examined. Heyes and colleagues [61,62], on the other hand, advocated an associative sequence learning theory. Their theory does not suppose an inherent ability to detect self–other equivalence and instead employs postnatal mapping between one’s own actions and those of others. Our model, which is based on predictive learning, bridges the gap between these contradicting hypotheses. It learns postnatally the correspondence between the self and others while leveraging the similarity between them that is emphasized by perceptual development. We thus suggest that our theory provides a unified computational account for the emergence of the mirror neuron system.

### (b) Hierarchical development of goal-directed actions

The second example is the development of goal-directed actions, which also appears through the process of learning the predictor as shown in figure 2*a*. Behavioural studies reported that infants exhibit a hierarchical representation of actions during imitation [37–39]. If the goal of an action is salient, infants tend to imitate only the goal while ignoring the means (i.e. how to achieve the goal). If the goal state is not underlined or the means are highlighted conversely, they reproduce the whole process of an action. These findings indicate that the goal in most actions has a higher priority than the means owing to its saliency; thus, the goal tends to be selectively imitated by infants.

We hypothesized that differences in prediction error for the goal and means produce such hierarchical representation of actions [57]. The goal, which is defined as the difference between the initial and final states, involves the largest change in an action; thus, the discrepancy between the goal and a current state produces the largest prediction error. By contrast, the means exhibits a smaller prediction error than the goal. As it corresponds to the sequential states through the whole action, its discrepancy from the current state appears smaller than the goal. This gap in the prediction error is hypothesized to lead to the hierarchical development of actions. A larger prediction error for the goal would be minimized first, and thus only the goal is imitated in early infancy. Then, a smaller error for the means is minimized after the error for the goal becomes small enough, resulting in the development of the whole action. Although other factors such as perceptual development in infants might also affect their performance of imitation, influences of such factors can consistently be accounted for by prediction error.

**Figure 5.**
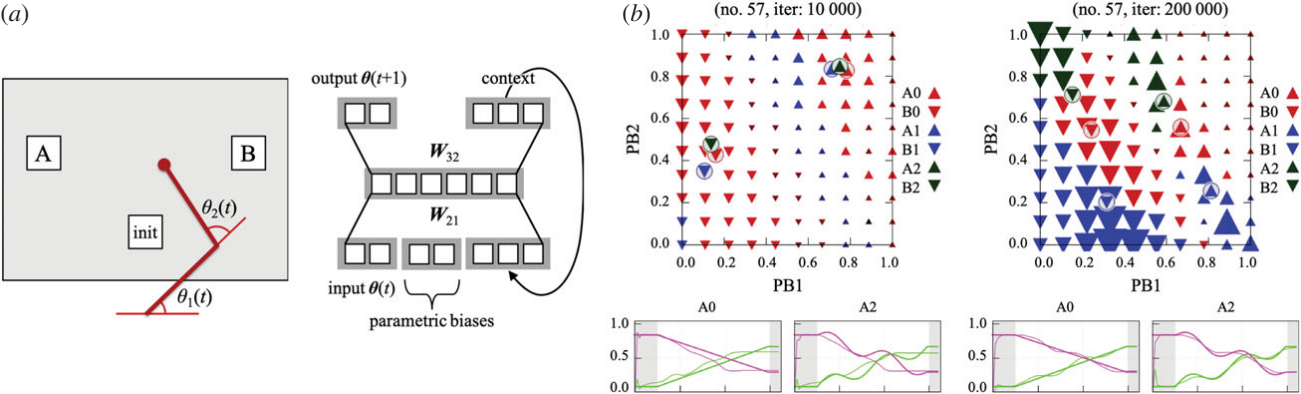
Hierarchical development of goal-directed actions based on predictive learning (adapted from [57]). (*a*) An experimental setting for a reaching task (left) and a recurrent neural network used as a predictor (right). A two-link arm robot learned to reach for a target (A or B) by following one of three predefined trajectories (0: straight, 1: sinusoidal curve, 2: sinusoidal curve with double frequency). The network learned temporal sequences of sensorimotor signals by minimizing prediction error. (*b*) Analysis of the parametric biases (top) and the output of the network (bottom). After 10 000 iterations of training (left), the network reproduced only the goals but not the means. Two clusters in the PB space show the internal representation corresponding to the generated actions. Only the network after 200 000 iterations of training (right) accurately reproduced the actions by properly differentiating parametric biases. (Online version in colour.)

To verify the above hypothesis, we designed a reaching task for a two-link arm robot as shown in figure 5*a* (left) [57]. The task was to move the end effector of the robot from the initial position (init) to a target position (A or B) by following one of three predefined trajectories (0: straight trajectory, 1: sinusoidal curve, 2: sinusoidal curve with double frequency). There were thus six types of actions as the combinations of the two goals and three means. For the predictor of the robot, we adopted a recurrent neural network with parametric bias (RNNPB), as drawn in figure 5*a* (right). An RNNPB has the ability to learn multiple time series of signals by adopting the parametric bias (PB) [26]. The values for the PB are self-organized to differentiate multiple sequences through learning, while the connecting weights of the network are updated to minimize prediction error. Note that the goal and means do not need to be separately coded in the RNNPB, but their relative difference in the prediction error leads to the hierarchical development. For the current experiment, only the joint angles of the robot (*θ*_1_(*t*) and *θ*_2_(*t*)) were used as the motor and proprioceptive signals. Sensory signals from other modalities (e.g. vision) can be integrated as additional input/output signals, which would not affect our hypothesis. The simplified setting allowed us to systematically analyse the developmental process observed in the RNNPB.

Figure 5*b* shows the learning process of the six actions: the self-organized PB values (top) and the output of the network (bottom) after 10 000 (left) and 200 000 (right) training iterations. A close analysis of PB values enabled us to investigate the internal representation of the actions. The most important finding is that the RNNPB exhibited hierarchical development of actions, as observed in infants. After 10 000 iterations of training, the RNNPB differentiated only the goals but not yet the means. As seen in figure 5*b* (left), the six circles with a triangle inside separated only into two groups: (A0, A1, A2) on the right and (B0, B1, B2) on the left. The first group corresponded to the actions directed to the goal A, whereas the second corresponded to the actions directed to B. The output of the RNNPB (A0 and A2 as examples) also reproduced only the goal of the actions but not the trajectories yet. After 200 000 iterations of training, the RNNPB finally differentiated the six actions as shown in figure 5*b* (right). The six circles separated from each other while maintaining the relationship between the goal-relevant clusters. The output of the network accurately imitated both the goal and means. This result suggests that predictive learning reproduces not only resultant behaviours but also their developmental dynamics observed in infants.

### (c) Emergence of helping behaviour

The third example is the emergence of helping behaviour. In contrast to the previous two studies, which focused on the process of updating the predictor, this experiment demonstrated how a predicted motor signal can be used to generate primary social behaviour such as helping (i.e. the second mechanism shown in figure 2*b*). Developmental studies revealed that helping action is observed in 14-month-old infants [63,64]. For example, if an experimenter drops a clothespin on the floor while hanging a towel, infants spontaneously approach the clothespin and hand it over to the experimenter. Importantly, infants show this kind of prosocial behaviour even without receiving any request (e.g. gesture and speech) or reward for their help. An open question was what motivates infants to help others.

**Figure 6.**
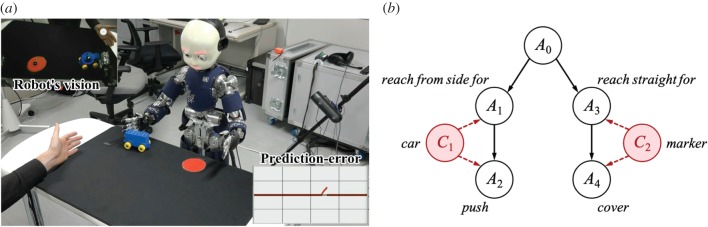
Emergence of helping behaviour based on predictive learning (adapted from [58]). (*a*) An experimental setting, in which a robot is pushing a car on behalf of a person because the car is beyond the person’s reach. An increase in prediction error (shown in the lower right corner) triggers the execution of the robot’s action to minimize it. (*b*) A predictor the robot acquired in the setting shown in (*a*). Two types of actions (i.e. push a car and cover a marker) are represented using a probabilistic model, where A and C denote actions and sensory states, respectively. (Online version in colour.)

We proposed a computational model based on predictive learning [58]. Our key idea was that the process of minimizing prediction error by executing a predicted action results in helping behaviour. Figure 6*a* shows the experimental setup, where the robot first learned to push a blue car and to cover a red marker by itself. Figure 6*b* depicts the acquired predictor designed by a probabilistic model. The nodes $A=(A_{0},A_{1},\ldots ,A_{4})$ and $C=(C_{1},C_{2})$ denote actions and sensory conditions, respectively. In this experiment, all the actions (e.g. reach for and push) were predesigned, and the goals of the actions were defined as the final state of action sequences. The probabilistic model indicates that the robot learned to move a car by first reaching for its side (*A*_1_) and then pushing it (*A*_2_), and to hide a marker by first reaching straight for it (*A*_3_) and then covering it (*A*_4_). Once the predictor was acquired, it could be applied to anticipate others’ actions. If other individuals successfully performed actions, the prediction error remained small and thus no action was executed by the robot. Only if they failed to achieve actions was an increase in the prediction error detected, which triggered an execution of the robot’s action. In the scene shown in figure 6*a*, an experimenter tried to push the car by extending his arm but failed to achieve it as the car was beyond his reach. Then the robot, which was observing the experimenter, detected an increase in the prediction error as shown in the lower right corner of figure 6*a* and was propelled to execute its anticipated action to minimize the error, which resulted in helping the experimenter.

The reason for the robot being triggered to minimize the error, although the action was originally performed by another person, is the mirror neuron system. As described before, the mirror neuron system is hypothesized to develop as a byproduct of predictive learning. Therefore, the robot in the early development, which cannot yet differentiate the self from others, tries to minimize prediction error regardless of the original actor. Such undifferentiated representation, however, makes it difficult for the robot to take another person’s perspective. The robot, for example, would pull a car towards its body instead of pushing it towards a person if the person tries to pull it towards himself. The issue of perspective taking has not been addressed in our current model, and it is known in developmental psychology that this ability develops later in infancy [65]. Although further studies to address this issue are necessary, our current result supports our hypothesis that the development of proto-social behaviour originates in the minimization of prediction error.

Psychologists have proposed two hypotheses to account for possible motivations for infant help [66]. The first hypothesis is called emotion sharing, which assumes infants’ ability to infer others’ intention and feeling (i.e. unobservable state). If infants observe failure in other people's actions, they try to please them by helping. The second hypothesis is called goal-alignment, which presupposes infants’ ability to recognize others’ action and its outcome (i.e. observable state). In this view, infants would take over someone else's goal as if it were the infants’ own goal if the other persons do not produce an expected result. Our theory based on predictive learning is analogous to the second hypothesis and moreover provides a biologically plausible account for the neural mechanism. In the future we aim to demonstrate more varieties of helping behaviours based on this hypothesis.

## 4. Autism spectrum condition caused by atypical tolerance for prediction error

The robot experiments have demonstrated the roles of predictive learning in cognitive development. However, they addressed only typical patterns of development, and behavioural diversity such as developmental disorders has not been discussed yet. This section presents our current hypothesis to account for ASC by extending the predictive learning theory.

ASC is a neurodevelopmental disorder, which is characterized by difficulties in social communication and preference for repetitive behaviours [49–51]. People with ASC show weaker abilities to follow other people's gaze, to read others’ intention and emotion, etc. In addition to traditional research focusing on social behaviour, recent studies have been investigating atypical perception in ASC [67–69]. Examples of their findings include hypersensitivity/hyposensitivity [70,71] and local processing bias [72] in perception, which are viewed as potential causes for behavioural characteristics of ASC. Frith and Happé [73,74] and Ayaya and Kumagaya [75,76] hypothesize that the way of precessing sensorimotor signals differs between ASC and typical development and thus causes communication difficulties between them. The brain is supposed to integrate incoming signals to recognize the world and to interact with it. It is suggested, however, that individuals with ASC have a diminished ability to integrate signals and/or a hyper-ability to process primitive signals, which results in dysfunctions in higher social cognition [73–75].

**Figure 7.**
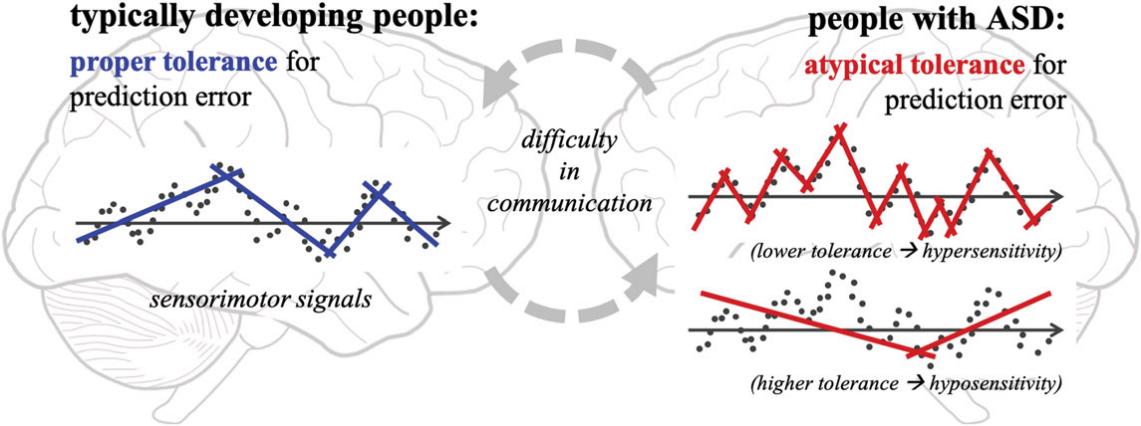
Potential underlying mechanism for ASC. Typically developing people (left) apply a proper tolerance for the prediction error and thus acquire appropriate internal models with adaptability (multiple lines in the left graph). People with ASC (right), on the other hand, adopt a lower or a higher tolerance for the prediction error and thus obtain strictly or loosely fitting models without adaptability or reactivity, respectively (the upper and lower graphs in the right part). Our hypothesis suggests that such a difference in their internal models causes difficulties in social interaction. (Online version in colour.)

Inspired by the above hypothesis, we propose a computational account for the underlying mechanism of ASC. Figure 7 illustrates a conceptual model based on predictive learning. Let us assume that sensorimotor signals denoted by data points on the graphs are fed into the brain, and the internal model (i.e. the predictor) represented by lines is acquired by linear regression for the sake of simplicity. Here, the internal model includes any type and any level of cognition (e.g. motor control and perception) and is characterized by multiple properties such as hierarchy, accuracy, and so on. According to [75], people with ASC seem to have atypical tolerance for prediction error. Figure 7*a* shows the internal model of typically developing (hereafter TD) people. They adopt a proper tolerance for prediction error and thus acquire an adequate internal model to perceive and act in the environment. Note that the model represented by multiple lines loosely fits the data points, indicating that TD people can easily adapt to environmental changes. In contrast, people with ASC obtain different internal models from those acquired by TD people. Figure 7*b* shows two types of atypicality: a lower tolerance (the upper graph) and a higher tolerance (the lower graph) for predictive learning. A lower tolerance, on the one hand, generates a strictly fitting model with less adaptability. People with a lower tolerance are expected to be very sensitive to environmental changes because they are always surprised by changes and need to update their internal model to properly perceive the world. Such a characteristic would appear not only in their motor control but also in perception, so-called perceptual hypersensitivity. A higher tolerance, on the other hand, results in a loosely fitting model with less reactivity. People with a higher tolerance may not realize small changes in the environment and thus exhibit seeking behaviours for stronger stimuli. Their perceptual characteristic is viewed as hyposensitivity, which is another type of atypical perception.

Similar hypotheses have been proposed in the past few years. Pellicano & Burr [77] presented a Bayesian explanation for ASC. Humans are thought to perceive the world as a consequence of inference based on sensory signals and priors. Their Bayesian account argues that individuals with ASC recognize the world more accurately and are less biased by previous experiences because of hypo-priors. This hypothesis was then interpreted from the viewpoint of predictive coding [78–80]. People with ASC and without ASC share the same mechanism of predictive learning; however, those with ASC are hypothesized to have less strong prediction and/or higher weight for prediction error, which results in precise characteristics in ASC. Our hypothesis, compared to the above, addresses the two aspects of ASC, i.e. hypersensitivity and hyposensitivity. The former corresponds to the precision account investigated intensively in previous studies, whereas the latter is an opposite phenomenon less discussed before. A recent study suggests that hypersensitivity and hyposensitivity should be considered as two sides of the same coin [75]. One can exhibit both hypersensitivity and hyposensitivity simultaneously, which implies that a coherent explanation that accounts for contradicting characteristics is necessary. Our hypothesis takes this point into account and provides a comprehensive explanation of ASC.

Another important suggestion here is that difficulties in social communication appear *between* TD and ASC, but not *within* ASC. Because people with and without ASC do not share internal models, they cannot easily predict each other's behaviours or intentions. In other words, TD people exhibit a lack of theory of mind towards ASC, as people with ASC do towards TD. This idea provides us with a new perspective; people are expected to be able to improve communication if they share internal models with some kind of help. To address this issue, we have been developing a head-mounted display system to simulate atypical perception in ASC [81,82]. We analysed visual experiences of ASC and revealed causal relationships between audiovisual stimuli from the environment and atypical visual patterns perceived by ASC (e.g. higher contrast, lower acuity and dotted noise). Our simulator reproducing the causal relationships enables TD people to experience the visual world of ASC and thus share, although not exactly, internal models with ASC. We also identified neural and physiological evidence that supports our findings. Atypical perception seems to be produced not only at sensory levels but also at signal processing levels in the brain [82]. We aim to investigate our hypothesis further by designing computational models of ASC.

## 5. Discussion

Sections 3 and 4 presented case studies of robot experiments and an extended theory for ASC. They demonstrate important roles of predictive learning in cognitive development but leave some open questions. This section summarizes our contributions and discusses open issues for further refinement of the theory.

### (a) Neural correlates of predictive learning

Recent studies in neuroscience have reported that multiple brain areas respond to prediction error detected in sensorimotor signals. Summerfield & Koechlin [83], for example, revealed that the inferior temporal gyrus shows increased activation in response to visual prediction error. Apps *et al.* [84] found neural activities related to multimodal (visual-tactile) prediction error in the inferior temporal gyrus and the temporo-parietal junction. They suggest that prediction error minimization plays an important role in multimodal integration required for self-cognition [85]. Blakemore *et al.* [86] examined neural activities involving action. They found that the somatosensory cortex activates differently to a self-produced tickle and to an externally produced one. The tactile sensation in the former condition is more predictable (i.e. with lower prediction error) and thus induces weaker neural activation, whereas the sensation in the latter condition evokes stronger neural response owing to its lower predictability (i.e. higher prediction error). All these findings indicate the involvement of the sensorimotor cortex in predictive coding.

Our theory and computational models have not specified brain regions correlated to the predictor. Rather we have intended to model the predictor as a whole of the sensorimotor cortex. A reason is our focus on the process of cognitive development; our goal is to understand the underlying mechanism for development during early infancy. It makes us assume that functions of the infant brain are not yet clearly differentiated, unlike those in the brain of adults. For example, an imaging study of newborns shows activation of broader brain areas induced by tactile stimuli [87]. Although our current models are very simple, in the future we plan to investigate how functionalities of the brain as well as cognitive abilities develop through sensorimotor predictive learning.

There is also debate on sensory prediction versus motor prediction as functions of the human brain. Wolpert *et al.* [20], for example, suggest the prediction of motor signals as a function of the brain. Friston *et al.* [21,22], in contrast, explain the fundamental mechanism as sensory prediction. To my knowledge, a comprehensive conclusion has not yet been reached. Our current model involves both sensory and motor prediction, and motor prediction plays important roles in explaining the development of mirror neuron systems and proto-social behaviours. Further investigations in neuroscience and computational modelling will be necessary to answer this question.

### (b) Developmental origin of predictive learning

How the function of predictive learning *per se* emerges based on dynamical neural activities is also an important question. Although behavioural evidence from young infants suggests the potential existence of such an ability in their brain, it is still controversial whether the ability is inherently embedded or acquired through learning during, for example, the fetal period. Recent advances in modelling of large-scale neural networks (e.g. [88]) have a potential to provide insights into the developmental origin of predictive learning. Simulating microscopic neural activities allows researchers to investigate what kinds of neural functions emerge from the neural dynamics and how sensorimotor experiences influence the functional development. A dynamic neural network model embodied in a fetus simulator [89] would also contribute greatly to uncovering the role of sensorimotor learning during the fetal period. Our future work includes integrating their findings into our theory to thoroughly explain development.

### (c) Towards a unified theory for cognitive development

The greatest contribution of our study is to have verified the key roles of predictive learning in cognitive development. Psychologists have presented many theories and hypotheses to explain their findings but separately for different behaviours. To my knowledge, only a few theories exist, such as the dynamical systems approach [1,2], which can consistently account for infant development. Many existing studies in cognitive developmental robotics [3–5] have also proposed task-specific architectures for different behaviours. Although their computational models nicely demonstrated infant-like development in robots, the underlying mechanisms for the continuity and diversity in development have not well been explained. By contrast, our approach has partially overcome these problems; we have demonstrated that multiple developmental phenomena can be attributed to predictive learning. Besides the case studies presented in this paper, we have shown how the abilities of intention reading [48,90], emotion sharing [91] and preferential looking [92] develop based on predictive learning.

A neural network model to verify the hypothesis about ASC has also been proposed [93]. Our latest results suggest that modifying model parameters related to the tolerance for prediction error produces different types of internal models, which correspond to TD and ASC. Moreover, ASCs with hypersensitivity or hyposensitivity can be reproduced with two extremes of model parameters. Nevertheless, we admit that our theory about ASC is still speculative and that further studies integrating neuroscience, cognitive psychology and computational modelling are necessary. For example, questions such as how differently (or similarly) the brain with or without ASC responds to prediction error, what types of prediction error the brain detects, and how differences in the neural activity affect behaviours should be addressed. Although several studies have addressed these issues, they show either neural or behavioural evidence that is not well linked yet. We believe that a computational approach such as presented in the paper has a potential to bridge the gap between neuroscience and cognitive psychology. Further studies tightly linking these approaches should be conducted to verify this hypothesis.

## 6. Conclusion

This paper has presented a computational theory for early cognitive development. We have emphasized the crucial role of predictive learning in multiple cognitive functions. Recent studies in neuroscience and cognitive science suggest that the human brain codes sensorimotor signals in a predictive manner. We extended this view and proposed two mechanisms that lead to cognitive development, i.e. updating an immature predictor and executing a predicted action. Our experiments using robots clarified the roles of predictive learning in early cognitive development. The first mechanism of predictive learning enabled a robot to recognize the self and other individuals based on their predictabilities, and to learn to produce goal-directed actions. Importantly, the function of the mirror neuron system emerged as a byproduct of this development. The second mechanism enabled a robot to produce primary social behaviours. By exploiting the mirror neuron system acquired in the predictor, the robot produced imitative actions and helping behaviour in response to actions generated by others.

Our future challenge is to investigate to what extent the predictive learning theory accounts for cognitive development. In terms of diversity of development, we presented our current hypothesis about ASC. Atypical tolerance for prediction error provides a plausible explanation for their perceptual and behavioural characteristics. The conceptual model shown in figure 7 provides a good illustration as to why people with and without ASC have difficulties in social interaction. However, there is neural and physiological evidence regarding atypicality in sensory and brain structures in ASC (e.g. [94–96]). Our hypothesis cannot yet explain what neural structures are correlated with atypical function. It is also controversial whether atypicality in the sensory and brain structures leads to atypicality in their function or *vice versa*. To obtain better insights into the underlying mechanism, we aim to further investigate the influence of atypical tolerance on predictive learning.

In terms of continuity of development, we have thus far addressed cognitive behaviours appearing from the neonatal period to early infancy. We do not know yet whether higher social cognition such as language use and decision making is also acquired by predictive learning. Our speculation is that such cognitive abilities require hierarchical representation of sensorimotor signals using symbols. Inhibitory control would also be necessary for explaining actions that do not always minimize prediction error. Reward-based learning may give another important criterion for development. We thus aim at extending our theory to incorporate higher cognition.

## Data accessibility

This article has no additional data.
